# SETAC GLB and SETAC Europe SAC: a liaison promoting the next generation of ecotoxicologists and environmental chemists

**DOI:** 10.1186/s12302-018-0171-z

**Published:** 2018-10-31

**Authors:** Simon Lüderwald, Kymberly Newton, Katharina Heye, Kristina Bitter, Samuel Moeris, Lena Benner, Paul Böhm, Josef Koch, Alexander Feckler, Mafalda Castro, Andreas Erikkson

**Affiliations:** 1 Institute for Environmental Sciences, University of Koblenz, Landau, Germany; 2 Department of Biological Sciences, University of Montreal, Montreal, Canada; 3 Department Aquatic Ecotoxicology, Goethe University, Frankfurt am Main, Germany; 4 Institute for Environmental Research, RWTH, Aachen, Germany; 5 Department of Animal Sciences and Aquatic Ecology, Ghent University, Ghent, Belgium; 6 Department of Aquatic Sciences and Assessment, Swedish University of Agricultural Sciences, Uppsala, Sweden; 7 Department of Analytical Chemistry and Environmental Sciences (ACES), Stockholm University, Stockholm, Sweden; 8 Department of Biological and Environmental Science, University of Jyväskylä, Jyväskylä, Finland

## Abstract

This commentary is an introduction for students to the Society of Environmental Toxicology and Chemistry (SETAC) and its Student Advisory Council (SAC). As young academics face challenges while trying to develop their careers, SETAC and the SAC help facilitate student involvement in the various communities within the society that can help to develop the students’ careers within the environmental sciences [e.g. the German Language Branch (GLB)]. This piece would also like to emphasize and pay homage to the continual cooperation between the SAC and the ESEU, which provides a scientific platform to communicate internationally and beyond the borders of SETAC, as well as offer heartfelt congratulations from the SAC to the GLB for their “20 Years SETAC GLB” and deep gratitude for their strong advocacy and support of the SAC.

## A solution to the riddle

How do you know what you want to be when you grow up? Some people know from the time they are very little that they want to be Doctors, Architects or Firemen and they continue on this path for the rest of their lives. For most of us, however, that is not the case. Even after deciding on a study program, the hunt is not over. Let us say you have decided on environmental sciences and are approaching the end of your graduate studies with an ecotoxicology or environmental chemistry focus. What are you going to do with your degree and how do you develop a career path after graduation?

As a young academic faced with these challenges, getting involved with SETAC (the Society of Environmental Toxicology and Chemistry) is the right place to start. Whether you are interested in continuing in academia or are considering a government or industry career, participating in SETAC meetings will help you to meet the right people and to get started achieving your career goals. While it might be intimidating at first to attend Annual Meetings of SETAC’s Geographic Units or even a World Meeting with all these experts in your field around, SETAC also has a way of helping introduce young scientists into the Society. The SETAC Europe Student Advisory Council (SAC), consisting of ten active students—currently affiliated with Goethe University Frankfurt, Germany; Stockholm University, Sweden; University of Jyväskylä, Finland; Ghent University, Belgium; University of Koblenz-Landau, Germany; and RWTH Aachen, Germany—represents SETAC Europe’s student members (approximately one-fourth of the whole society).

The SAC was established in 2006 as an initiative of Prof. Dr. Ralf Schulz (University of Koblenz-Landau, Germany) and Dr. Fred Heimbach (formerly Bayer CropScience). Their goal was to improve communication among students within SETAC and to create opportunities to meet senior scientists. Originally an ad-hoc group of three Ph.D. students (Mirco Bundschuh, University of Koblenz-Landau, Germany; Amy Brooks, Sheffield University, United Kingdom; and Thomas-Benjamin Seiler, University of Heidelberg, Germany), the SAC developed into a profound part of SETAC Europe.

In addition to facilitating connections between student members, the SAC regularly organizes student activities during the Annual Meetings of SETAC Europe, such as Career Talks and Mentor Lunches to bring together students and established scientists. On top of the student events at the Annual Meetings, the SAC also organizes the Young Environmental Scientists (YES) Meeting, along with SETAC North America’s Student Advisory Council (NASAC), annually altering the venues between Europe and North America. The YES Meeting is a conference organized by students for students, at which participants have the opportunity to give platform or poster presentations in a relaxed environment of their peers without paying any conference fees. While the Society, and both the Annual Meeting and the YES Meeting offer great opportunities to get involved, in the beginning, it can still be a little overwhelming to present your research in a second language.

For German-speaking students, the SETAC Europe’s German Language Branch (GLB) offers a solution to this problem, as they are provided with the opportunity to attend the Annual GLB Meeting and present their research in the comfort of their own language. Therefore, the GLB’s Annual Meeting offers students and young scientists an easy and comparatively inexpensive way to get in contact with other scientists of related fields, prospective employers, international activities and the SETAC network. In fact, a number of present and former members of the SAC started presenting their research and getting involved in SETAC at a GLB Annual Meeting. As one of the GLB’s main goals is to promote young scientists in the area of environmental sciences, it also offers prestigious awards to the students who wrote the best master and doctoral theses each year. Furthermore, the SETAC GLB is affiliated with Environmental Science Europe (ESEU); a journal featuring a ‘SETAC GLB Corner’, which among other things, provides a communication platform for the SAC, allowing to reach out to an audience beyond the borders of SETAC.

Since the foundation of the SAC, SETAC GLB has always been a strong advocate and supporter of the SAC and associated events like the YES meeting. As the SETAC GLB approaches its 20th anniversary the SAC offers sincere gratitude and congratulations to the GLB upon its celebration of “20 Years SETAC GLB” and looks forward to a productive and fruitful future collaboration.

